# Diversity and Composition of Microbial Communities in an Eelgrass (*Zostera marina*) Bed in Tokyo Bay, Japan

**DOI:** 10.1264/jsme2.ME21037

**Published:** 2021-10-13

**Authors:** Md Mehedi Iqbal, Masahiko Nishimura, Md. Nurul Haider, Masayoshi Sano, Minoru Ijichi, Kazuhiro Kogure, Susumu Yoshizawa

**Affiliations:** 1 Atmosphere and Ocean Research Institute, The University of Tokyo, 5–1–5 Kashiwanoha, Kashiwa, Chiba 277–8564, Japan; 2 Department of Natural Environmental Studies, Graduate School of Frontier Sciences, The University of Tokyo, 5–1–5 Kashiwanoha, Kashiwa, Chiba 277–8563, Japan; 3 Department of Fisheries Technology, Faculty of Fisheries, Bangladesh Agricultural University, Mymensingh, Bangladesh; 4 National Institute of Polar Research, 10–3 Midori-cho, Tachikawa, Tokyo 190–8518, Japan

**Keywords:** *Zostera marina*, 16S rRNA, eelgrass microbiome, dead leaf, Tokyo Bay

## Abstract

*Zostera marina* (eelgrass) is a widespread seagrass species that forms diverse and productive habitats along coast lines throughout much of the northern hemisphere. The present study investigated the microbial consortia of *Z. marina* growing at Futtsu clam-digging beach, Chiba prefecture, Japan. The following environmental samples were collected: sediment, seawater, plant leaves, and the root-rhizome. Sediment and seawater samples were obtained from three sampling points: inside, outside, and at the marginal point of the eelgrass bed. The microbial composition of each sample was analyzed using 16S ribosomal gene amplicon sequencing. Microbial communities on the dead (withered) leaf surface markedly differed from those in sediment, but were similar to those in seawater. Eelgrass leaves and surrounding seawater were dominated by the bacterial taxa *Rhodobacterales* (*Alphaproteobacteria*), whereas *Rhodobacterales* were a minor group in eelgrass sediment. Additionally, we speculated that the order *Sphingomonadales* (*Alphaproteobacteria*) acts as a major degrader during the decomposition process and constantly degrades eelgrass leaves, which then spread into the surrounding seawater. Withered eelgrass leaves did not accumulate on the surface sediment because they were transported out of the eelgrass bed by wind and residual currents unique to the central part of Tokyo Bay.

*Zostera marina* (eelgrass) is a widespread seagrass species that generates ecological diversity and economically important ecosystems along coast lines throughout much of the northern hemisphere (Marba *et al.*, 2006; Duffy *et al.*, 2015). Seagrass habitats are considered one of the most valuable marine ecosystems on earth (Costanza *et al.*, 1997; Hemminga and Duarte, 2000) because they support marine life, including epiphytic organisms as well as coastal fisheries resources (Coles *et al.*, 1993; Hemminga and Duarte, 2000), and contribute to marine environments by stabilizing bottom sediment and maintaining coastal water quality (Komatsu and Yamano, 2000). Highly productive seagrass habitats play an important role in producing organic matter from carbon dioxide (Meteo *et al.*, 2006), and are involved in the sequestration of organic carbon through the dying and collapsing process. However, massive declines in seagrass beds have occurred worldwide due to human-induced environmental deterioration (Orth *et al.*, 2006; Waycott *et al.*, 2009). Seagrass beds (particularly *Z. marina*) around the coastal areas of Japan have declined since the 1960s, mainly due to land reclamation (Aioi and Nakaoka, 2003).

The eelgrass body is generally made up of two main parts: leaves and the root-rhizome. The leaf part stays underwater, whereas the root part is embedded in water-saturated sediment. Each plant part associates with its unique bacteria (Ettinger *et al.*, 2017; Fahimipour *et al.*, 2017), thereby carving out another ecological niche for these bacteria. Eelgrass leaves start to decompose from the tip, become fragmented, wash out, and may be exported far away from the meadow before settling on sediments (Gallagher *et al.*, 1984; Bach *et al.*, 1986). The decomposition of *Z. marina* leaf detritus fuels a number of food webs near to and distant from the beds (Hemminga and Duarte, 2000). Litter and particulate detritus derived from vascular plants are biologically processed mainly via detrital food webs based on microbial decomposition (Valiela *et al.*, 1985; Benner *et al.*, 1988; Mann, 1988). Once dead leaves flow into the surrounding environment, they may increase the diversity of microbial flora.

In recent years, an extensive amount of research has focused on microbial communities associated with terrestrial plants. The health and function of land plants are often maintained by their associated microbiomes (Berendsen *et al.*, 2012; Vorholt, 2012; Turner *et al.*, 2013). In contrast to land plants, limited information is currently available on the microbes associated with aquatic angiosperms, such as seagrass. Recent culture-independent studies have focused on relationships with general sediment bacteria in the seagrass rhizosphere (Cifuentes *et al.*, 2000; James *et al.*, 2006; Green-García and Engel, 2012; Sun *et al.*, 2015; Cúcio *et al.*, 2016; Zhang *et al.*, 2020), epiphytic microbes on *Hallophila stipulacea* (Mejia *et al.*, 2016), and comparisons of seagrass microbes with those of surrounding environments in *Z. marina*, *Zostera Japonica*, *Zostera muelleri* (Ettinger *et al.*, 2017; Fahimipour *et al.*, 2017; Crump *et al.*, 2018; Hurtado-McCormick *et al.*, 2019), *Thalassia testudinum*, and *Syringodium filliforme* beds (Ugarelli *et al.*, 2019; Vogel *et al.*, 2020). The majority of community analyses revealed that the taxonomical composition of microbial communities varied among seagrass tissues, namely, leaves and the root-rhizome (Crump and Koch, 2008; Mejia *et al.*, 2016; Ettinger *et al.*, 2017; Fahimipour *et al.*, 2017). Furthermore, the composition of microbial communities differed between seagrass bodies and the surrounding environments (Jensen *et al.*, 2007; Cúcio *et al.*, 2016; Mejia *et al.*, 2016; Crump *et al.*, 2018; Hurtado-McCormick *et al.*, 2019; Vogel *et al.*, 2020). In contrast, a recent study on eelgrass microbiomes showed that leaf communities appeared to be similar to those of the surrounding seawater (Fahimipour *et al.*, 2017). Moreover, Hurtado-McCormick *et al.* (2020) reported that the *Z. muelleri* seagrass microbiome varied by the type of leaf pigmentation (Hurtado-McCormick *et al.*, 2020). These findings are crucial for understanding seagrass-microbe interactions and prompted us to conduct this research.

Since eelgrass grows thickly and decays on a yearly basis, it may scatter not only a large amount of leaf litter, but also particular microbial groups that densely accumulate on decayed leaves during the decomposition process of plant matter.

In the present study, we investigated the microbial consortia of *Z. marina* growing at Futtsu clam-digging beach, Chiba prefecture, Japan. We collected the following environmental samples: sediment, seawater, green healthy leaves, withered (dead) leaves, and the root-rhizome. The microbial communities of each sample were characterized using 16S ribosomal gene amplicon sequencing. The present study aimed to show similarities and differences in the microbial composition of different plant parts (*i.e.*, healthy and dead leaves and the root-rhizome) of eelgrass and the surrounding environments (*i.e.*, seawater, sediment) in order to obtain a more detailed understanding of the relationship between eelgrass host and surrounding environment microbial communities.

## Materials and Methods

### Site description and sample collection

Test samples were collected from eelgrass (*Z. marina*) beds growing at Futtsu clam-digging beach (35°18′56.91″N, 139°47′42.03″E), Chiba prefecture, Japan in the summer (July 05, 2016; Table 1).

Sediment and seawater samples were collected inside (10–15‍ ‍m from the margin), at the marginal area (within the eelgrass habitat), and outside (15–20‍ ‍m away from the margin) of the eelgrass bed for microbiome analyses. Sediment cores and seawater samples were collected from all sampling points (inside, outside, and at the margin) and plant bodies were cut out from eelgrass colonies (more detail see Table S1). Water samples were collected in a sterile plastic bottle (1 L) from the surface (0.1–0.5 m) layer and filtered through a Sterivex filter unit with a pore size of 0.22‍ ‍μm (Millipore) using a peristaltic pump. The sediment core was collected using a 50-mL plastic syringe. Water depth was approximately 0.5–1.0 m. Sediment core samples were collected in duplicate from each sampling point (inside, at the margin, and outside) and sediment cores were sectioned from the bottom surface at 5-cm intervals. Every core tube was sealed at both ends with a rubber stopper and immediately placed on ice.

Eelgrass plants were randomly selected and dug up by hand (including at least 5‍ ‍cm of the root components) at each sampling point. The plant bodies were then gently washed with the same water to remove sediment particles from the roots. To remove loosely-associated microbes and plankton, the plant surface was rinsed with filtered and autoclaved seawater (Weidner *et al.*, 1996; Burke *et al.*, 2009). Plant bodies were then cut into two parts: aboveground components (trunk and leaves) and belowground components (the rooting zone). Eelgrass blades were checked by a visual inspection and separated into two parts: greenish-healthy leaves and brownish-dead leaves (withered leaves). All samples were transported to the laboratory within 2 h of collection under chilled conditions. In the laboratory, each sediment core was cut into five vertical sections (the top 5‍ ‍cm, 1‍ ‍cm/section). Sediment and plant samples were frozen at –80°C for further analyses.

### Environmental variables and sediment properties

Temperature, pH, salinity, and dissolved oxygen were measured at approximately 20‍ ‍cm above the sediment using the Multi-parameter Water Quality Checker (U series; HORIBA). Sediment grain size was measured by laser diffraction spectroscopy (SALD-3000S; Shimazu). A part of the sediment samples was dried in a heating oven and then mechanically ground. Ground sediment samples were treated with 1 N HCl, rinsed with distilled water, dried, and packed in tin capsules. Sediment-bound total organic carbon (TOC) was measured using an elemental analyzer (FLASH 2000; Thermo Scientific).

### Nucleic acid extraction and purification

DNA was extracted from all samples (more detail see Table S1) using a FastDNA^®^ SPIN Kit for soil (MP Biomedical) according to the manufacturer’s instructions with some modifications. Bead beating was performed using a Micro Smash (MS-100R; Tomy Medico) at 2,000‍ ‍rpm and 4°C for 40 s.

Regarding water samples, Sterivex filters were manually excised and removed from the plastic holder. The filter removed from each holder was then transferred into a bead-beating tube (MS-100R; Tomy Medico). After thawing the frozen sediment core, 0.25‍ ‍g of sediment was added to another bead-beating tube. DNA extraction from eelgrass parts (the leaves and rooting zone) was performed according to Burke *et al.* (2009) with a slight modification. Briefly, 5‍ ‍g of each sample was placed into 15‍ ‍mL of washing solution (200‍ ‍mM Tris–HCl pH 8, 10‍ ‍mM EDTA, and 0.24% Triton X-100; Kadivar and Stapleton, 2003; Mejia *et al.*, 2016), incubated at 25°C for 1 h on a rotating disk (80‍ ‍rpm), and gently stirred with a vortex mixer. The supernatant was transferred into a new tube, and the same procedure was repeated twice for each sample. Forty-five milliliters of the supernatant was centrifuged at 300×*g* for 15‍ ‍min to remove plant residues. The supernatant was concentrated using the 30K Amicon ultra-15 centrifugal filter device (Merck Millipore). DNA extraction from the concentrated sample was performed using the FastDNA^®^ SPIN Kit for soil (MP Biomedical) according to the manufacturer’s instructions.

Extracted DNA was finally cleaned with the NucleoSpin (MACHEREY-NAGEL GmbH & Co. KG) gDNA Clean-up kit according to the manufacturer’s instructions and stored at –30°C until further use.

### Amplification of the 16S rRNA gene and pyrosequencing

The small ribosomal subunit (hypervariable V3–V4 region) of the 16S rRNA gene was amplified by a polymerase chain reaction (PCR) using the forward primer 342F with multiplex identifiers (MIDs): 5′-CCATCTCATCCCTGCGTGTCTCCGACTCAGXXXXXXXXXXCTACGGGGGGCAGCAG-3′ and the reverse primer 806R with an adaptor: 5′-CCTATCCCCTGTGTGCCTTGGCAGTCTCAG(GGACTACCGGGGTATCT-3′; where X represents the sample-specific MID (Mori *et al.*, 2013). PCR was performed in a volume of 20‍ ‍μL containing the following: 2‍ ‍μL (1‍ ‍ng μL^–1^) of the DNA template, 12.7‍ ‍μL of molecular biological grade double-distilled water, 0.8‍ ‍μL (5‍ ‍μM) of each primer, 2‍ ‍μL of 10× TaKaRa Ex Taq Buffer, 1.6‍ ‍μL of TaKaRa dNTP mixture (2.5‍ ‍mM each), and 0.1‍ ‍μL of TaKaRa Ex Taq HS Polymerase (TaKaRa) in triplicate. Thermal cycling was performed for 25 cycles under the following conditions: initial denaturation at 94°C for 4‍ ‍min, denaturation at 98°C for 10‍ ‍s, annealing at 55°C for 30‍ ‍s, elongation at 72°C for 1‍ ‍min, and final elongation at 72°C for 10‍ ‍min. After amplification, the presence of a PCR product was confirmed using agarose gel electrophoresis. PCR products were further purified and normalized using Agencourt AMPure XP (Beckman Coulter) according to the guidance of the 454 Sequencing Amplicon Library Preparation Method Manual (GS Junior Titanium Series 2012). PCR products were then quantified using the Quantifluor^®^ dsDNA System kit (Promega) and sequenced using the 454 GS Junior sequencer (Roche Diagnostics, 454 Life Sciences) according to the manufacturer’s instructions for the 454 GS Junior Titanium Series.

### Sequence data accession number

Raw sequence data are available at the DDBJ Sequence Read Archive (DRA) database under the accession number DRA010365.

### Sequence processing

Subsequent analyses, including quality checks and arrangements, were conducted as described by Haider *et al.* (2018) using the open-sourced MOTHUR program (Schloss *et al.*, 2009) following the operation manual for 454 (http://www.mothur.org/wiki/454_SOP). Each unique sequence was initially aligned with align.seqs using the SILVA reference (silva.nr_v128) alignment (Pruesse *et al.*, 2007). The pre-cluster method (Huse *et al.*, 2010) was then applied to reduce sequencing errors by screening, filtering, and de-noising. Chimeric sequences were identified and removed using chimera.uchime (Edgar *et al.*, 2011). Sequences were subsequently classified using the database of the Ribosomal Data Project (Maidak *et al.*, 1996). Inactive components, such as chloroplasts, mitochondria, and organelles affiliated with “former” bacterial sequences, were removed from our dataset. High-quality sequences were used to generate a distance matrix and clustered assigning to operational taxonomic units (OTUs) at the 97% identity level (Schloss and Westcott, 2011). A representative sequence from every OTU was used for classification by running the MOTHUR program based on SILVA reference (silva.nr_v128) databases. To standardize the number of sequences between samples, they were randomly re-sampled to the sample with the fewest reads (3,570 reads) using the MOTHUR program based on the OTU files clustered at a cut-off level of 0.03.

### Data visualization and statistical analyses

Data visualization and statistical analyses were performed exclusively in R software (R Core Team, 2020) with the Phyloseq (McMurdie and Holmes, 2013), vegan (Oksanen *et al.*, 2020), and ggplot2 (Wickham, 2016) packages. Chao1 (Chao *et al.*, 2005) and Shannon indices (Shannon and Weaver, 1949) were used to calculate intra-sample diversity. To assess the inter-sample diversities of microbial communities associated with different sample groups (including the sample type and sampling point), non-metric multidimensional scaling (NMDS) was performed using Bray-Curtis (Bray and Curtis, 1957) dissimilarities based on the normalized (rarified) OTU abundance data of each sample. The permutation test was performed via the “adonis” function of the “vegan” package (Oksanen *et al.*, 2020). The DESeq2 package (Love *et al.*, 2014) was used to detect pairwise differences in taxonomic abundance based on the seagrass tissue type (healthy leaf vs root & rhizome). *P* values were adjusted using the false discovery rate (FDR) correction method (Benjamini and Hochberg, 1995) and a log2 fold change plot was made with significant OTUs at *P*<0.05. An analysis of variance (ANOVA) was also performed in R to test the significance of differences among groups for alpha diversity matrices, after which Tukey’s *post-hoc* Honest Significant Difference (HSD) test (Tukey, 1953) was applied to test pairwise group differences. To assess whether environmental factors significantly varied between sampling points in the seagrass bed, we initially performed the Kruskal-Wallis test, followed by Dunn’s test with Bonferroni corrections to test pairwise group differences.

## Results

### Environmental variables and sediment properties of the studied area

The environmental parameters and sediment properties of sampling points (inside, at the margin, and outside) are shown in Table 1. Significant differences were observed in temperature, salinity, and dissolved oxygen among the sampling points (inside, at the margin, and outside; Kruskal-Wallis, *P*<0.01). Sediment grain sizes and TOC in the sediment also significantly differed among the sampling points (Kruskal-Wallis, *P*<0.01; Table 1). Grain sizes inside and outside were 216.4 and 248.6‍ ‍μm, respectively, and were all classified as fine sand according to Wentworth’s size classification (Wentworth, 1922). Grain sizes were significantly smaller in the vegetated area (inside) (Dunn’s test with Bonferroni corrections, *P*<0.01) than in the unvegetated area (outside; Table 1).

### Microbial compositions of eelgrass beds and surrounding areas

To profile microbial communities from *Z. marina* healthy green leaves, dead (withered) leaves, the rooting zone (root & rhizome), eelgrass sediment, seawater surrounding eelgrass, and unvegetated samples (*i.e.*, sediment and seawater), we performed high throughput 454 pyrosequencing. We obtained 168,680 sequences consisting of 17,647 phylotypes (OTUs) from sequencing data (Fig. S1, the rarefaction curve showed the number of OTUs obtained against the total number of sequences for each sample).

**Fig. 1.**
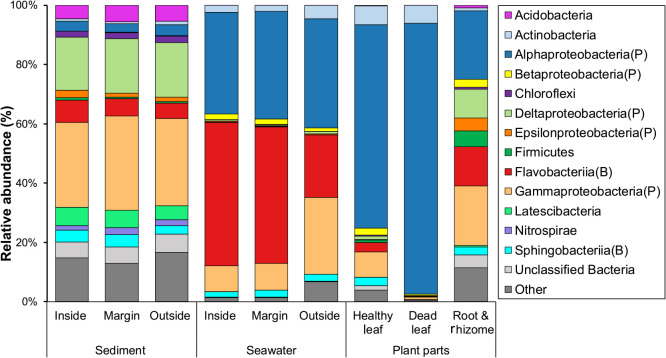
Microbial community compositions of seagrass beds and unvegetated (outside) environments *i.e.*, seawater and sediment shown at the phylum level. Two phyla, *Proteobacteria* and *Bacteroidetes*, are shown at the class level. Each letter in brackets represents a respective phylum name: P, *Proteobacteria*; B, *Bacteroidetes*. Relative abundance is shown as a mean value calculated from at least two replicates of each sample. Phyla that contributed less than 2% were combined and referred to as “Others”. The groups without a classification were referred to as “Unclassified Bacteria”.

Microbial communities associated with *Z. marina* and those of the surrounding area samples are shown at the highest assigned taxonomic level in Fig. 1. The major classes of important phyla, *Proteobacteria* and *Bacteroidetes*, were separately shown. The phyla that contributed to less than 2% of the total abundance were combined and referred to as “Others” and those with no affiliation as “Unclassified”. *Proteobacteria* and *Bacteroidetes* were the dominant microbial groups in eelgrass bed samples (leaves, the rooting zone, eelgrass sediment, and seawater surrounding eelgrass).

The microbial communities of vegetated areas (inside and at the margin) were compared with those of unvegetated areas (outside; Fig. S2). The classes *Flavobacteriia* (47.2±6.5%; Mean±SD), *Alphaproteobacteria* (35.3±3.9%), and *Gammaproteobacteria* (8.8±1.9%) were dominant in seawater surrounding eelgrass (inside and at the margin), while *Alphaproteobacteria* (36.8±1.3%), *Gammaproteobacteria* (25.9±0.7%), and *Flavobacteriia* (20.9±0.9%) were dominant in unvegetated seawater (outside; Table S2 and Fig. 1). However, no significant differences were observed in the seawater microbial composition between vegetated areas (inside and at the margin; Table S2, Fig. 1 and S2A).

The classes *Gammaproteobacteria* (30.2±3.2%) and *Deltaproteobacteria* (18.2±4.3%) were dominant in eelgrass sediment (inside and at the margin). Other major groups were affiliated with the classes *Flavobacteriia* (6.7±2.3%), *Latescibacteria* (5.9±1.6%), and *Sphingobacteriia* (4.0±1.1%; Table S2 and Fig. 1). The frequently-appearing taxa of eelgrass sediment (inside and at the margin) were similar to those of unvegetated sediment (outside); however, slight differences were observed in the relative abundance of major taxa between the three sediment samples (Table S2, Fig. 1 and S2B).

*Alphaproteobacteria* (68.6±28.0%) and *Gammaproteobacteria* (8.6±5.8%) were the dominant classes of epiphytic bacteria associated with green healthy leaves. Other taxa were affiliated with *Acidimicrobiia* (5.1±1.8%) and *Flavobacteriia* (3.2±2.3%; Table S2 and Fig. 1). *Alphaproteobacteria* were also the most dominant (91.4±2.5%) on the surface of brownish dead leaves. Other groups appearing on dead leaves were affiliated with *Actinobacteria* (4.1±1.2%) (Table S2 and Fig. 1).

Although there was no predominant class among rooting zone (root & rhizome) microorganisms, different classes were detected, such as *Alphaproteobacteria* (23.1±2.4%), *Gammaproteobacteria* (20.1±2.8%), *Flavobacteriia* (13.2±2.4%), *Deltaproteobacteria* (9.7±3.1%), *Clostridia* (Firmicutes) (5.3±1.5%), and *Epsilonproteobacteria* (4.3±1.5%; Table S2 and Fig. 1).

Moreover, several rare groups were found in eelgrass samples, including *Acidobacteria*, *Chloroflexi*, and *Nitrospirae* (Table S2 and Fig. 1).

### Comparison of microbial communities among eelgrass and surrounding environment samples

A differential abundance analysis identified 24 OTUs with significant fold changes between healthy leaves and root & rhizome samples: twelve each from healthy leaves and root & rhizome samples (DESeq2 Benjamini–Hochberg corrected *P<0.05*, Fig. 3). This analysis identified several genera belonging to the class *Alphaproteobacteria* (*e.g. Jannaschia*, *Marivita*, *Sulfitobacter*, and *Sphingomonas*) that were dominant on healthy leaves, whereas other genera belonging to the classes *Epsilonproteobacteria* (*Arcobacter*) and *Deltaproteobacteria* (*e.g. Desulforhopalus* and *Desulfobacula*) were enriched on the root-rhizome (Fig. 3).

**Fig. 2.**
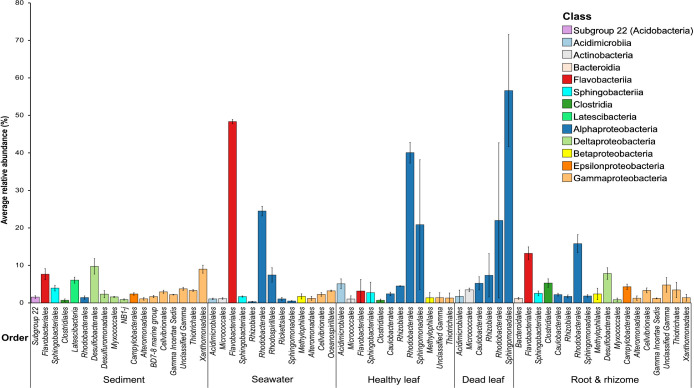
Relative abundance of major microbial taxa associated with each sample type (eelgrass sediment, seawater surrounding eelgrass, healthy leaves, dead leaves, and root & rhizome). OTUs were grouped into taxonomic categories (order), and each column was color-coded according to the taxonomic class. Orders with a mean abundance of one percent (≥1%) are shown. Bars represent the standard error of the mean.

We also compared microbial communities in green healthy and brownish dead leaves. On dead leaves, the major groups were affiliated with *Sphingomonadales* and *Rhodobacterales* (Fig. 2). They were also found on healthy leaves. However, the order *Sphingomonadales* (particularly the genus *Sphingomonas*; Fig. S3) was significantly more dominant on dead leaves than on healthy leaves. Leaf (healthy and dead) communities were also compared to those of the surrounding environments *i.e.*, seawater and sediment. The order *Rhodobacterales* (*Alphaproteobacteria*) was a minor group in eelgrass sediment (1.4%.); however, it significantly contributed to the relative abundance of bacteria associated with greenish healthy leaves (40.0%) and brownish dead leaves (22.0%) as well as bacteria in seawater surrounding eelgrass (24.5%; Table S2 and Fig. 2).

### Intra-sample (alpha diversity) variations between sample types and sampling points

Alpha diversity was evaluated in terms of species richness and diversity using the Chao 1 and Shannon diversity indices. The alpha diversity metrics of three different points were compared (Fig. 4A). Alpha diversity values were markedly higher for sediment samples than for water samples (ANOVA, Tukey’s test, Shannon, *P*<0.001; Chao1, *P*<0.001; Fig. 4B). Among seawater samples, diversity values were higher for unvegetated areas (outside) than for vegetated areas (inside and at the margin; ANOVA, Shannon, *P*<0.01; Chao1, *P*<0.05; Fig. 4A). However, no significant differences were observed in alpha diversity values between inside and margin samples (ANOVA, Tukey’s test, Shannon, *P*=0.54; Chao1, *P*=0.81; Fig. 4A). Microbial diversity was also compared between sample types: eelgrass sediment, seawater surrounding eelgrass, healthy leaves, dead leaves, and the rooting zone (Fig. 4B). Alpha diversity values were greater in sediment, followed by the rooting zone, healthy leaves, seawater, and dead leaves (ANOVA, Shannon, *P*<0.001; Chao1, *P*<0.001; Fig. 4B). No significant differences were observed in alpha diversity values between leaf samples and surrounding water samples (ANOVA, Tukey’s test, *P*>0.05; Fig. 4B), with the exception of the Shannon index between dead leaves and surrounding seawater (ANOVA, Tukey’s test, Shannon, *P*=0.02). However, a significant difference was observed in alpha diversity values between eelgrass sediment and dead leaves (ANOVA, Tukey’s test, Shannon, *P*<0.001; Chao1, *P*<0.001; Fig. 4B). Therefore, the alpha diversity of dead leaves appeared to be more similar to that of surrounding seawater than to that of eelgrass sediment.

### Inter-sample variation (beta diversity) between sample types and sampling points

Based on the relative abundance of appearing microbial groups, NMDS was generated using Bray-Curtis dissimilarity and used to represent sample coordinates on a two-dimensional perceptual map. The NMDS plot in Fig. 5 shows the communities clustered by sample types as well as sampling points (inside, at the margin, and outside). The PERMANOVA test indicated that these clusters significantly differed among sample types (*R^2^*=0.64, *P*=0.001; Fig. 5) and slightly differed among sampling points (seawater, *R^2^*=0.72 and *P*=0.06; sediment, *R^2^*=0.11, *P*=0.09; Fig. 5). However, a pairwise PERMANOVA test showed a significant difference in the microbial composition between eelgrass sediment and leaf samples (pairwise PERMANOVA; *P*<0.05; Table S3), but not between surrounding seawater and leaf samples (pairwise PERMANOVA; *P*>0.05; Table S3)

## Discussion

The main aim of the present study was to elucidate the role of each microbial community on marine plants by characterizing the microbial communities associated with different plant parts (*i.e.*, healthy leaves, dead leaves, and the rooting zone) and comparing them to those of the surrounding environments *i.e.*, seawater and sediment of the eelgrass bed. Previous studies on the eelgrass microbiome mainly focused on healthy leaf tissue and comparisons with those of the surrounding environments (Ettinger *et al.*, 2017; Fahimipour *et al.*, 2017; Crump *et al.*, 2018). However, a comprehensive analysis of the epiphytic microbial community in different leaf tissues (*i.e.*, healthy and dead leaves) and their relationship with the surrounding environment has not been conducted in sufficient detail to discuss the role of each microbial community. Therefore, the present study also aimed to provide a basis for understanding the microbial processes involved in eelgrass degradation by clarifying the microbial communities of each plant part and the surrounding environment. The results obtained showed that leaf (healthy and dead) surface microbial communities markedly differed from those of sediment, but were similar to those of seawater. Furthermore, members of the class *Alphaproteobacteria* significantly contributed to the relative abundance of bacteria associated with leaf tissue *i.e.*, healthy leaves, dead leaves, and bacteria in seawater surrounding eelgrass; however, they were one of the minor groups in the eelgrass sediment. These results suggest that the class *Alphaproteobacteria* may colonize the seawater surrounding eelgrass leaves, degrade them, and spread them into the surrounding seawater.

The beta diversity analysis showed distinct communities in the surrounding environments (*i.e.*, seawater and sediment) as well as in seagrass samples (including the leaves and roots). These results are consistent with previous findings showing differences in microbial compositions not only between leaves and the rooting zone (Crump and Koch, 2008; Ettinger *et al.*, 2017; Fahimipour *et al.*, 2017), but also between seagrass and the surrounding environment (Jensen *et al.*, 2007; Gordon-Bradley *et al.*, 2014; Cúcio *et al.*, 2016; Mejia *et al.*, 2016; Crump *et al.*, 2018; Hurtado-McCormick *et al.*, 2019; Vogel *et al.*, 2020). The classes *Alphaproteobacteria* and *Gammaproteobacteria* were dominant on the surface of *Z. marina* green healthy leaves (Table S2 and Fig. 1). The class *Alphaproteobacteria* accounted for 68% of the total community on healthy leaves. These results are consistent with the findings reported by Mejia *et al.* (2016) showing that the epiphytic bacteria of *H. stipulacea* leaves mainly comprised the class *Alphaproteobacteria*, followed by the class *Gammaproteobacteria*. Crump and Koch (2008) demonstrated that *Z. marina* leaves were dominated by *Alphaproteobacteria* (Crump and Koch, 2008). The present results also revealed that the order *Rhodobacterales* were enriched on *Z. marina* healthy leaves (Fig. 2). *Rhodobacterales* are purple non-sulfur bacteria that are often surface attached in marine habitats and have the ability to fix nitrogen (Palacios and Newton, 2005; Dang *et al.*, 2008; Ettinger *et al.*, 2017). This group has constantly been detected on *Z. marina* leaves (Ettinger *et al.*, 2017) as well as on *H. stipulacea* leaves (Weidner *et al.*, 2000; Mejia *et al.*, 2016) and the tropical seagrass *Thalassia hemprichii* (Jiang *et al.*, 2015). In the present study, the order *Rhodobacterales* (*Alphaproteobacteria*) also significantly contributed to the relative abundance of bacteria associated with *Z. marina* dead leaves as well as bacteria in the seawater surrounding eelgrass (inside and at the margin). The present results indicate that the members of this group play an important ecological role in association with eelgrass.

The pairwise PERMANOVA test showed similar microbial compositions in leaf and surrounding seawater samples. Furthermore, leaf alpha diversities revealed values close to those of the surrounding seawater, with the exception of dead leaves in Shannon (Fig. 4B). These results are consistent with the recent findings of a global study on *Z. marina* microbiomes (Fahimipour *et al.*, 2017). In this global study, the composition of the leaf microbes of eelgrass was similar to that of the surrounding seawater and a source tracking analysis suggested that surrounding seawater was the primary source of colonists for eelgrass leaves (Fahimipour *et al.*, 2017). Therefore, we assume that leaf microbes have a close relationship with the water layer around the eelgrass bed. In the present study, the leaf microbiome of *Z. marina* at the class level showed similarities with the microbiomes in the surrounding seawater, with *Alphaproteobacteria* being the most abundant class in both cases. However, these similarities were not apparent at the order level. As an example, the order *Flavobacteriales* were dominant in the surrounding seawater, but were a minor group on the eelgrass leaf (Fig. 2). In addition, in comparisons with outside seawater, seawater surrounding eelgrass (inside and at the margin) was significantly dominated by *Flavobacteriales* (Fig. S2). Members of the class *Flavobacteria* are important decomposers of high-molecular-weight organic matter, such as cellulose (Kirchman, 2002; Fenchel, 2012), which implies the abundance of this group in the eelgrass bed. *Sphingomonadales* (*Alphaproteobacteria*), particularly the genus *Sphingomonas*, was significantly more abundant on dead leaves than on healthy leaves (Fig. 2 and S3). Previous studies also confirmed the presence of this group on the surface of the marine macroalga *Ulva australis* (Burke *et al.*, 2011) and on the seaweed *Saccharina japonica* (Zhang *et al.*, 2020). Members of the phylum *Proteobacteria*, including the class *Alphaproteobacteria*, were the most abundant in the decomposition process of mangrove trees (Moitinho *et al.*, 2018). However, to the best of our knowledge, this is the first study to show the presence of *Sphingomonas* on the surface of the decaying leaves of *Z. marina*. Therefore, the genus *Sphingomonas* may act as a major degrader during the decomposition process of eelgrass; however, further environmental and experimental investigations are required to confirm this. Moreover, *Sphingomonas* are globally known for their unique ability to degrade large numbers of different organometallic compounds (Muangchinda *et al.*, 2015; Feng *et al.*, 2019; Asaf *et al.*, 2020) and are one of the important genera for carbon cycling (Wang *et al.*, 2021). The decomposition of eelgrass is followed by the production of a large amount of leaf litter, which diffuses into the surrounding water areas, scattering members of the class *Alphaproteobacteria* enriched on the dead leaf surface.

**Fig. 3.**
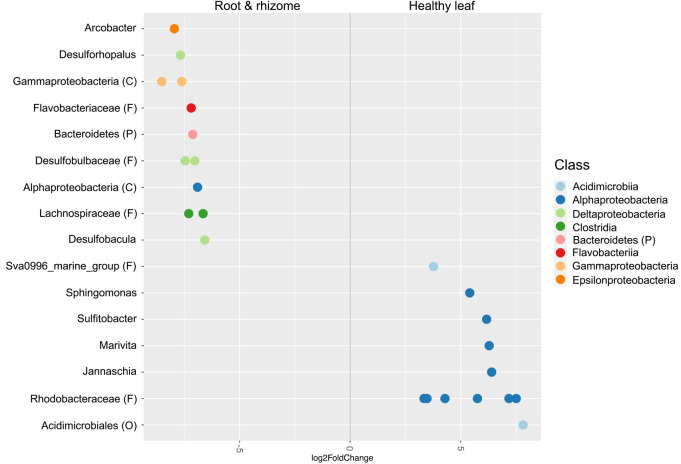
Pairwise comparison (DeSeq analysis). Differential abundant OTUs (*P<0.05*) on healthy leaf and root & rhizome counterpart samples are shown. OTUs were assigned to the genus level (y-axis) and class level (colors). OTUs not defined at the genus level were shown using the most specific taxonomic level available followed by a letter in brackets. Each letter represents a respective taxonomic level: P, Phylum, C, Class; O, Order; F, Family. Negative “log2 Fold Change” values (x-axis) indicate higher abundance on root & rhizome and positive values indicate higher abundance on healthy leaves.

The classes *Gammaproteobacteria*, *Deltaproteobacteria*, *Flavobacteriia*, and *Latescibacteria* were dominant in eelgrass sediment (Table S2 and Fig. 1), which was consistent with the recent findings of a culture-independent study on *Z. marina* microbiomes and rhizosphere microbiomes of three seagrasses, including *Z. marina* (Cúcio *et al.*, 2016; Ettinger *et al.*, 2017). The abundant classes were *Gammaproteobacteria*, *Deltaproteobacteria*, *Flavobacteriia*, and *Bacteriodia* in the rhizosphere sediment of the *Z. marina* bed (Cúcio *et al.*, 2016; Ettinger *et al.*, 2017). In the present study, rooting zone microorganisms comprised several classes, including *Alphaproteobacteria*, *Gammaproteobacteria*, *Flavobacteriia*, *Deltaproteobacteria*, *Clostridia*, and *Epsilonproteobacteria* (Table S2 and Fig. 1). Mejia *et al.* (2016) also reported that the classes *Gammaproteobacteria*, *Alphaproteobacteria*, and *Deltaproteobacteria* were dominant groups in *H. stipulacea* seagrass roots and rhizomes (Mejia *et al.*, 2016). A differential abundant analysis revealed that the Futtsu eelgrass rooting zone was dominated by the genera *Arcobacter* (*Epsilonproteobacteria*) and *Desulforhopalus* and *Desulfobacula* (*Deltaproteobacteria*; Fig. 3). This is further supported by recent studies on aquatic plant microbiology, in which microbial groups related to the sulfur cycle were predominantly found in *Z. marina* root samples (Jensen *et al.*, 2007; Ettinger *et al.*, 2017; Fahimipour *et al.*, 2017; Crump *et al.*, 2018). Sulfide accumulates to relatively high levels in eelgrass sediment as a result of sulfate reduction, which is highly toxic to eelgrass (Goodman *et al.*, 1995). Sulfur-oxidizing bacteria alleviate sulfide toxicity (Joshi and Hollis, 1977). The sulfur-oxidizing taxon *Arcobacter* (Wirsen *et al.*, 2002; Sievert *et al.*, 2007) may take part in environmental clean-up within the Futtsu eelgrass beds, and, thus, likely benefits eelgrass bodies, as has been shown in a previous study (Crump *et al.*, 2018). Sulfate-reducing bacteria have been identified as important contributors to nitrogen fixation in seagrass beds (Capone, 1982; Welsh, 2000; Nielsen *et al.*, 2001). Crump *et al.* (2018) reported that the species *Arcobacter nitrofigilis* dominated in the seagrass root (Crump *et al.*, 2018). This species has been reported as a nitrogen-fixing symbiont of salt marsh *Spartina alterofigilis* (Pati *et al.*, 2010).

**Fig. 4.**
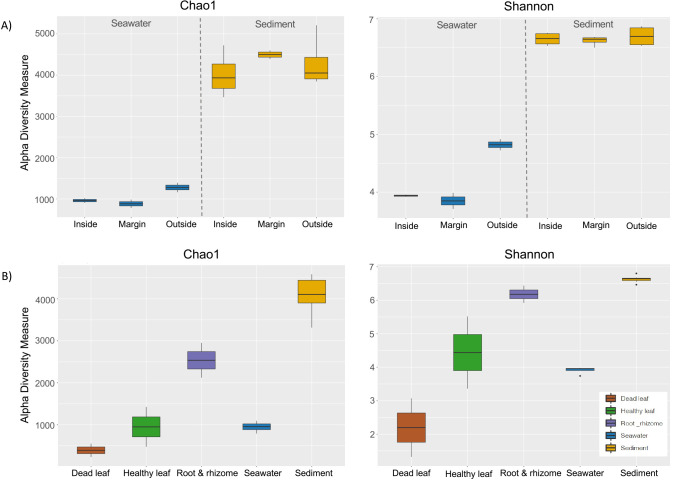
Alpha diversity of test samples. Two alpha diversity metrics, the Chao 1 and Shannon diversity indices are shown as boxplots for (A) sediment and seawater samples from three sampling points (inside, at the margin, and outside) and for (B) sample types (dead leaves, healthy leaves, root & rhizome, seawater surrounding eelgrass, and eelgrass sediment).

**Fig. 5.**
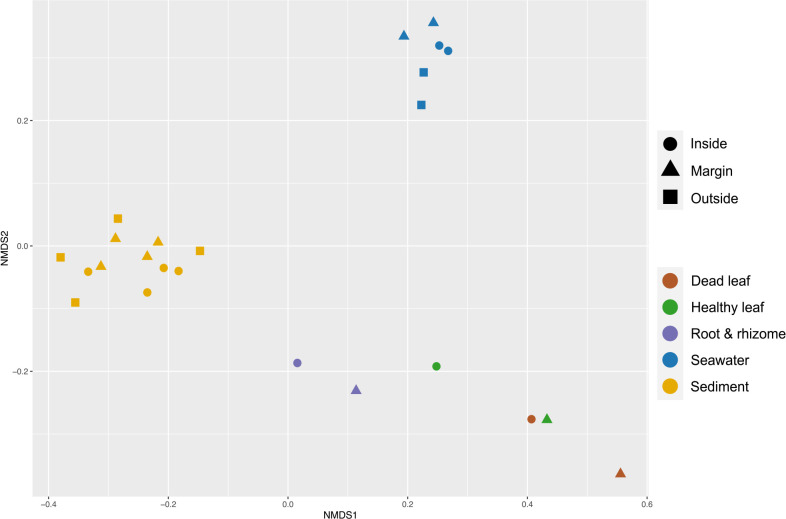
Non-metric multidimensional scaling (NMDS) based on Bray-Curtis distance matrices showing distinct clusters according to sample types and sampling points in the Futtsu seagrass area. Each sample was color-coded according to the sample type (dead leaf, healthy leaf, root & rhizome, seawater, and sediment) and plotted according to the sampling point (inside, at the margin, and outside).

Alpha diversity was high in the eelgrass sediment, followed by the rooting zone (Fig. 4B). Edwards *et al.* (2015) reported that rhizosphere soil gave rise to greater microbial diversity than other host-associated samples (including leaves and roots). Alpha diversity values for sediment markedly differed from those for seawater samples. Regarding seawater samples, diversity values were higher at the unvegetated area (outside) than at the vegetated area (inside and at the margin; Fig. 4A). Therefore, in contrast to expectations, the microbial diversity of eelgrass beds is not necessarily rich. Similar findings were obtained in a recent study on the microbial community structure in *Z. marina*-inhabited sediment (Ettinger *et al.*, 2017).

Aquatic bacteria are separated into two lifestyles: particle-associated and free-living. In aquatic environments, suspended particles are densely colonized by marine bacteria. These particles play an important role in transporting adherent bacteria into other object surfaces. Bacteria are presumed to move backward and forward between eelgrass beds and water columns through the process of particle adhesion. In the present study, microbial communities on dead leaves markedly differed from those of eelgrass sediment. Dead leaves also showed a significantly lower alpha diversity value than eelgrass sediment (Fig. 4B). Dead leaves were dominated by the class *Alphaproteobacteria* (order *Sphingomonadales* and *Rhodobacterales*), whereas *Alphaproteobacteria* were a minor group in eelgrass sediment (Table S2, Fig. 1 and 2). Since dead leaves decay and immediately get buried, a certain level of similarity may exist in microbial compositions between leaves laying above the sediment and in the bottom sediment just underneath. Based on these results, torn-off dead leaves may not be embedded in the bottom sediment, they appear to be transported out of the eelgrass bed by wind and residual currents unique the central part of Tokyo Bay (Guo and Yanagi, 1996). In previous studies (2015; unpublished), the physical and chemical properties of the following domestic eelgrass meadows were examined: Futtsu, Takehara (Ikuno-shima Is.), Nanao Bay, and Mutsu Bay (Table S4). In comparisons with other eelgrass meadows, Futtsu possessed a low-carbon and higher granular sediment, which implies its unique condition, namely, leaf litter is easily transported away by wind and/or water currents (Fig. S4).

Guo and Yanagi (1996) showed the formation of residual currents in Tokyo Bay during the four seasons. Wind-driven and tide-induced residual currents were calculated from hydraulic-observed data. They found that the north bank of Futtsu-Misaki was washed and swept by a clockwise circulation, the flow velocity of which may peak at approximately 5‍ ‍cm/s (Guo and Yanagi, 1996; Tanaka, 2001). Strong residual currents in the middle of Tokyo Bay, as demonstrated by Guo and Yanagi (1996), are one of the transport mechanisms dispersing decayed leaves or suspended particles to surrounding water areas. Therefore, withered eelgrass may be a carbon resource that may increase the diversification of microbial flora in Tokyo Bay.

## Conclusion

The present study clarified the microbial consortia of *Z. marina* growing at Futtsu clam-digging beach, Tokyo Bay. The microbial communities on the leaf (healthy and dead leaves) surface markedly differed from those in sediment, but were similar to those in seawater. The order *Rhodobacterales* (*Alphaproteobacteria*) significantly contributed to the relative abundance of bacteria associated with leaf tissue *i.e.*, healthy and dead leaves, as well as bacteria in seawater surrounding eelgrass; however, this microbial group sparsely colonized eelgrass sediment. We speculated that the order *Sphingomonadales* (*Alphaproteobacteria*) functions as a major degrader during the eelgrass decomposition process. *Sphingomonadales* in the surrounding seawater may colonize eelgrass leaves, degrade them during the growing season, and finally spread them into the surrounding seawater. Further investigations and culture treatments are required to confirm the involvement of this group in the decomposition process of eelgrass leaves.

## Citation

Iqbal, M. M., Nishimura, M., Haider, M.. N., Sano, M., Ijichi, M., Kogure, K., and Yoshizawa, S. (2021) Diversity and Composition of Microbial Communities in an Eelgrass (*Zostera marina*) Bed in Tokyo Bay, Japan. *Microbes Environ ***36**: ME21037.

https://doi.org/10.1264/jsme2.ME21037

## Supplementary Material

Supplementary Material

## Figures and Tables

**Table 1.** Environmental variables and sediment properties of sampling points (inside, at the margin, and outside of seagrass beds). Temperature, salinity, pH, and dissolved oxygen (DO) were measured approximately 20‍ ‍cm above the sediment.

Sampling area and date	Sampling point	Location coordinates	Collected sample	Temperature (°C)	Salinity (PSU)^2^	pH	DO (mg L^–1^)	Sediment TOC^3^ (mg g^–1^)	Sediment grain size^3^ (μm)
Futtsu, Chiba^1^ July-2016	Inside	35°18′56.91″N, 139°47′42.03″E	Sediment, water, plant part	25.0	32.1	8.10	11.71	0.60±0.02	216.4
	Margin	35°18′56.05″N, 139°47′39.01″E	Sediment, water, plant part	25.3	31.8	8.11	11.57	—	—
	Outside	35°18′56.48″N, 139°47′44.41″E	Sediment, water	23.5	33.2	8.15	10.26	0.73±0.10	248.6

1) Prefecture name, 2) PSU, Practical Salinity Unit, 3) Samples collected in May 2015. Sediment TOC values represent the mean of three replicates±standard deviation
