# Supplementary material for: Diversity and Composition of Microbial Communities in an Eelgrass (Zostera marina) Bed in Tokyo Bay, Japan
Source: Microbes Environ. 2021 Oct 13;36(4):ME21037. doi: 10.1264/jsme2.ME21037 (PMC8674447; doi:10.1264/jsme2.ME21037)
Supplement: Supplementary file 1 — Supplementary Material [file 36_21037_s1.pdf]

Supplementary data

Table S1. Test samples collected at each sampling point (inside, margin and outside of the seagrass beds).

Sample type	Sampling point	Collected field sample	Process in laboratory	Number of sample used for DNA extraction (N)
Sediment	Inside	2 core	4 samples (2×2; top & bottom)[1]	4
	Margin	2 core	4 samples (2×2; top & bottom)[1]	4
	Outside	2 core	4 samples (2×2; top & bottom)[1]	4
Seawater	Inside	2 rep. (1L/rep.)	2 samples (0.2 µm filter)[2]	2
	Margin	2 rep. (1L/rep.)	2 samples (0.2 µm filter)[2]	2
	Outside	2 rep. (1L/rep.)	2 samples (0.2 µm filter)[2]	2
Plant part	Inside	5- 6 plant	1sample (5g; healthy leaf)[3]	1
			1sample (5g; dead leaf)[3]	1
			1sample (5g; root-rhizome)[3]	1
	Margin	5- 6 plant	1sample (5g; healthy leaf)[3]	1
			1sample (5g; dead leaf)[3]	1
			1sample (5g; root-rhizome)[3]	1

1) A sediment core was cut into five vertical sections from the bottom surface to 5 cm deep (1cm/ section). The top layer (0-1 cm) and the bottom layer (4-5 cm) were used for microbial analysis. 2) One litter (1L) seawater was filtered with a 0.22-µm pore-sized Sterivex filter unit for each. 3) Five gram (5g) sample was cut off from all plant, collected from each sampling point.

Table S2. Average relative abundances of major microbial taxa (domain Archaea represent a single group) present in all *Z. marina* samples (eelgrass sediment, eelgrass surrounding seawater and plant parts: inside and margin) and unvegetated environments (sediment and seawater: outside). Values are means of at least two replicates of each sample.

					Sediment			Seawater			Plant parts		
Domains	Phylum	Class	Order	Family	Inside	Maginal	Outside	Inside	Maginal	Outside	Healthy leaf	Dead leaf	Root & rhizome
Archaea					0.11	0.29	0.46	0.00	0.00	1.50	0.00	0.00	0.07
Bacteria	Acidobacteria				4.61	5.57	5.36	0.03	0.01	0.04	0.17	0.08	0.94
		Holophagae			1.29	1.30	1.36	0.01	0.00	0.00	0.03	0.04	0.33
		Subgroup_17	Subgroup_17		0.33	0.33	0.28	0.00	0.00	0.01	0.00	0.00	0.17
		Subgroup_21	Subgroup_21		0.57	0.81	0.70	0.00	0.01	0.00	0.00	0.00	0.08
		Subgroup_22	Subgroup_22		1.57	2.08	2.19	0.01	0.00	0.01	0.00	0.00	0.06
		Other_Acidobacteria			0.85	1.05	0.83	0.01	0.00	0.03	0.13	0.04	0.30
	Actinobacteria				0.86	0.73	1.16	2.35	2.00	4.46	6.40	5.92	0.88
		Acidimicrobiia	Acidimicrobiales		0.75	0.57	1.04	1.08	1.08	3.63	5.13	1.76	0.80
		Actinobacteria	Micrococcales		0.04	0.05	0.02	1.26	0.92	0.81	1.19	4.14	0.07
		Other_Actinobacteria			0.07	0.11	0.10	0.00	0.00	0.02	0.07	0.01	0.02
	Unclassified_Bacteria				5.31	5.57	6.21	0.24	0.10	0.19	1.51	0.18	4.33
	Bacteroidetes				18.77	15.63	12.74	50.79	49.37	26.38	6.53	0.16	22.47
		Bacteroidetes_BD2-2			1.70	1.19	0.81	0.01	0.04	0.01	0.00	0.00	0.49
		Bacteroidia			1.62	1.10	0.73	0.14	0.07	0.08	0.01	0.00	1.59
		Cytophagia	Cytophagales		1.15	0.97	1.07	0.07	0.09	0.27	0.14	0.00	0.46
		Flavobacteriia			7.66	5.85	5.14	48.36	46.09	20.99	3.20	0.16	13.24
			Flavobacteriales		7.66	5.85	5.14	48.36	46.09	20.99	3.20	0.16	13.24
				Cryomorphaceae	0.55	0.32	0.32	6.87	10.07	3.11	0.07	0.08	0.45
				Flavobacteriaceae	6.96	5.44	4.71	38.94	33.68	15.54	2.88	0.08	12.53
				Other_Flavobacteriales	0.16	0.08	0.11	2.56	2.34	2.35	0.24	0.00	0.26
		Sphingobacteriia	Sphingobacteriales		3.97	4.15	2.86	1.71	2.40	2.23	2.77	0.00	2.58
		Other_Bacteroidetes			2.67	2.36	2.14	0.49	0.68	2.79	0.41	0.00	4.11
	Chloroflexi				1.94	2.02	2.16	0.00	0.06	0.08	0.28	0.00	0.63
		Anaerolineae			0.74	0.76	0.78	0.00	0.00	0.00	0.00	0.00	0.29
		Other_Chloroflexi			1.20	1.26	1.37	0.00	0.06	0.08	0.28	0.00	0.34
	Gemmatimonadetes				1.33	1.76	1.88	0.01	0.00	0.03	0.00	0.00	0.26
	Latescibacteria	Latescibacteria			6.12	5.80	4.63	0.01	0.06	0.04	0.00	0.00	0.46
	Planctomycetes				0.75	0.22	1.67	0.04	0.04	0.09	0.75	0.00	1.37
	Nitrospirae				1.59	2.37	2.04	0.00	0.02	0.02	0.01	0.00	0.07
	Proteobacteria				53.88	56.11	54.64	45.86	47.83	65.42	81.27	92.82	60.49
		Alphaproteobacteria			3.25	2.79	3.78	34.38	36.36	36.84	68.68	91.45	23.14
			Caulobacterales		0.51	0.22	0.30	0.12	0.12	0.15	2.44	5.26	2.22
			Rhizobiales		0.31	0.37	0.45	0.34	0.50	1.19	4.52	7.38	1.74
			Rhodobacterales		1.44	1.20	1.37	24.51	23.99	22.34	40.07	22.01	15.81
				Rhodobacteraceae	1.44	1.20	1.37	24.51	23.99	22.34	40.07	22.01	15.81
			Rhodospirillales		0.29	0.61	0.79	7.47	8.46	8.64	0.04	0.00	0.16
			Rickettsiales		0.06	0.05	0.07	1.08	2.61	2.90	0.09	0.06	0.07
			Sphingomonadales		0.41	0.22	0.27	0.52	0.29	1.20	20.88	56.63	1.87
			Other_Alphaproteobacteria		0.23	0.12	0.52	0.34	0.39	0.41	0.65	0.13	1.26
		Betaproteobacteria	Methylophilales		0.10	0.13	0.09	1.85	1.89	1.15	2.28	0.55	2.65
		Deltaproteobacteria			17.98	18.45	18.40	0.45	0.40	0.84	1.17	0.03	9.74
			Bdellovibrionales		0.52	0.54	0.75	0.16	0.08	0.17	0.46	0.00	0.36
			Desulfarculales		0.42	0.32	0.76	0.00	0.02	0.01	0.00	0.00	0.05
			Desulfobacterales		9.73	10.97	8.91	0.09	0.09	0.15	0.02	0.00	7.52
				Desulfobacteraceae	6.02	6.02	5.03	0.04	0.05	0.01	0.00	0.00	1.74
				Desulfobulbaceae	3.70	4.93	3.87	0.05	0.04	0.14	0.02	0.00	5.78
				Other_Desulfobacterales	0.00	0.02	0.01	0.00	0.00	0.00	0.00	0.00	0.00
			Desulfuromonadales		2.34	1.42	1.34	0.00	0.05	0.00	0.00	0.00	0.21
			Myxococcales		1.62	1.36	1.70	0.12	0.02	0.06	0.16	0.01	0.87
			NB1-j		0.92	1.41	1.76	0.00	0.02	0.01	0.03	0.00	0.05
			Syntrophobacterales		0.23	0.49	0.80	0.00	0.01	0.01	0.00	0.00	0.01
			Sva0485		0.98	0.84	0.97	0.00	0.00	0.02	0.00	0.00	0.06
			Other_Deltaproteobacteria		1.22	1.09	1.42	0.09	0.10	0.41	0.50	0.01	0.63
		Epsilonproteobacteria			2.45	1.33	1.56	0.25	0.18	0.22	0.16	0.03	4.34
			Campylobacterales		2.42	1.32	1.56	0.25	0.18	0.22	0.16	0.03	4.32
			Other_Epsilonproteobacteria		0.03	0.01	0.00	0.00	0.00	0.00	0.00	0.00	0.02
		Gammaproteobacteria			28.61	31.85	29.43	8.82	8.89	25.98	8.63	0.77	20.18
			Alteromonadales		1.11	1.19	0.65	1.24	0.43	2.44	0.59	0.13	1.28
			BD7-8_marine_group		1.72	2.96	2.57	0.00	0.00	0.06	0.01	0.00	0.02
			Cellvibrionales		2.99	2.72	3.69	2.28	2.60	9.75	0.28	0.00	3.37
			Chromatiales		0.57	0.60	0.71	0.01	0.00	0.01	0.38	0.00	0.44
			Gammaproteobacteria_Incertae_Sedis		2.22	1.97	2.01	0.11	0.13	0.16	0.20	0.00	1.23
			Unclassified_Gammaproteobacteria		3.78	4.02	4.11	0.36	0.43	1.19	1.41	0.06	4.81
			Oceanospirillales		0.49	0.46	0.80	3.23	4.22	10.68	0.05	0.00	0.85
			Thiotrichales		3.34	2.62	1.80	0.18	0.14	0.35	1.33	0.00	3.49
			Xanthomonadales		9.01	11.70	8.84	0.03	0.12	0.15	0.07	0.00	1.41
				JTB255_marine_benthic_gr	8.98	11.67	8.81	0.02	0.10	0.11	0.06	0.00	1.41
				Other_Xanthomonadales	0.03	0.03	0.03	0.00	0.01	0.00	0.01	0.00	0.00
			Other_Gammaproteobacteria		3.39	3.59	4.24	1.38	0.83	1.18	4.32	0.59	3.28
		Other_Proteobacteria			1.49	1.56	1.38	0.12	0.11	0.38	0.34	0.00	0.45
	Spirochaetae	Spirochaetes	Spirochaetales		0.61	0.51	0.72	0.00	0.00	0.03	0.00	0.00	0.75
	Firmicutes	Clostridia			0.73	0.47	0.57	0.16	0.12	0.12	0.79	0.26	5.32
	Other				3.39	2.95	5.76	0.52	0.38	1.59	2.31	0.58	1.96

Table S3. Pairwise-PERMANOVA based on Bray-Curtis dissimilarity matrices to evaluate variations in the microbial community composition between sample type. Significant p values are in bold.

Sample group (pairwise)	R-Square	p -Value
Sediment vs Seawater	0.694	**0.003**
Sediment vs Healthy leaf	0.501	**0.019**
Sediment vs Dead leaf	0.566	**0.019**
Sediment vs Root-rhizome	0.469	**0.022**
Seawater vs Healthy leaf	0.751	0.067
Seawater vs Dead leaf	0.846	0.067
Seawater vs Root-rhizome	0.798	0.067
Healthy leaf vs Dead leaf	0.423	0.333
Healthy leaf vs Root-rhizome	0.493	0.333
Dead leaf vs Root-rhizome	0.702	0.333

Table S4. Sediment properties of domestic eelgrass meadows: Futtsu, Takehara (Ikuno-shima Is.), Nanao Bay, and Mutsu Bay (unpublished data collected in 2015)

Sampling area and date	Location coordinates	Sediment TOC (mg/g)	Sediment Grain size (µm)
Futtsu, Chiba[1] May-2015	35°18'56.91"N, 139°47'42.03"E	0.60±0.02	216.4
Ikuno-shima Is., Hiroshima[1] June-2015	34°17'52.92"N, 132°55'02.91"E	9.92±0.82	20.3
Nanao Bay, Ishikawa[1] July-2015	37°05'10.21"N, 136°52'14.62"E	3.94±0.31	72.6
Mutsu Bay, Aomori[1] Aug-2015	40°51'58.06"N, 140°50'00.04"E	1.75±0.3	133.8

1) Prefecture name

Sediment TOC values represent the mean of three replicates ± standard deviation

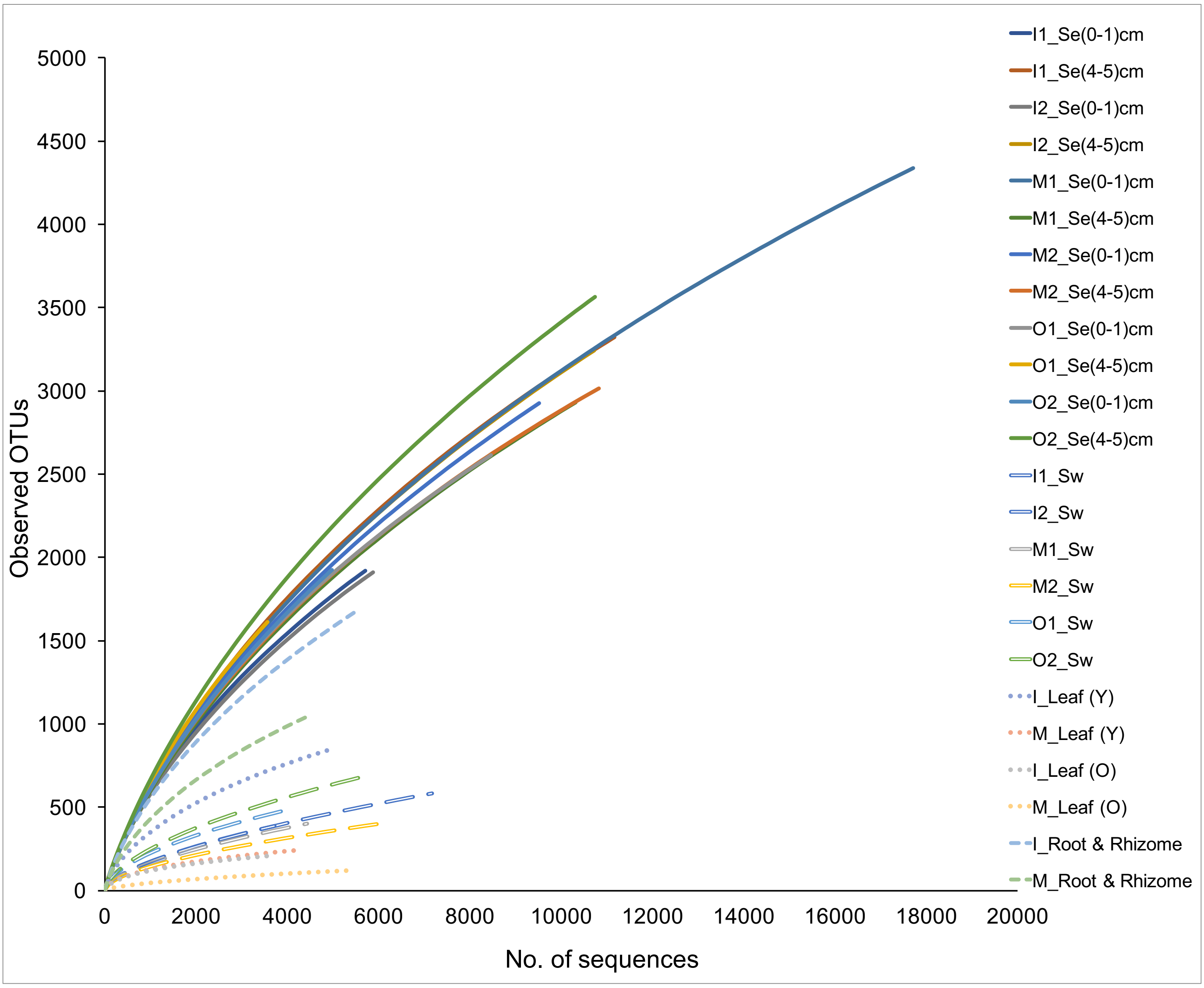

Fig. S1. Rarefaction curves of the all samples of the *Z. marina* seagrass bed (inside, margin) and unvegetated environment (outside), showing the number of observed phylotypes (OTUs) at 0.03 cut-off levels. Where the sample IDs' expressing; I, I1, I2=Inside seagrass bed; M, M1, M2=Margin of seagrass bed; O1, O2=Outside of seagrass bed; Se= Sediment; (0-1) cm= upper sediment; (4-5) cm= lower sediment; Sw= Seawater; (Y)= Healthy leaf; (O)= Dead leaf respectively.

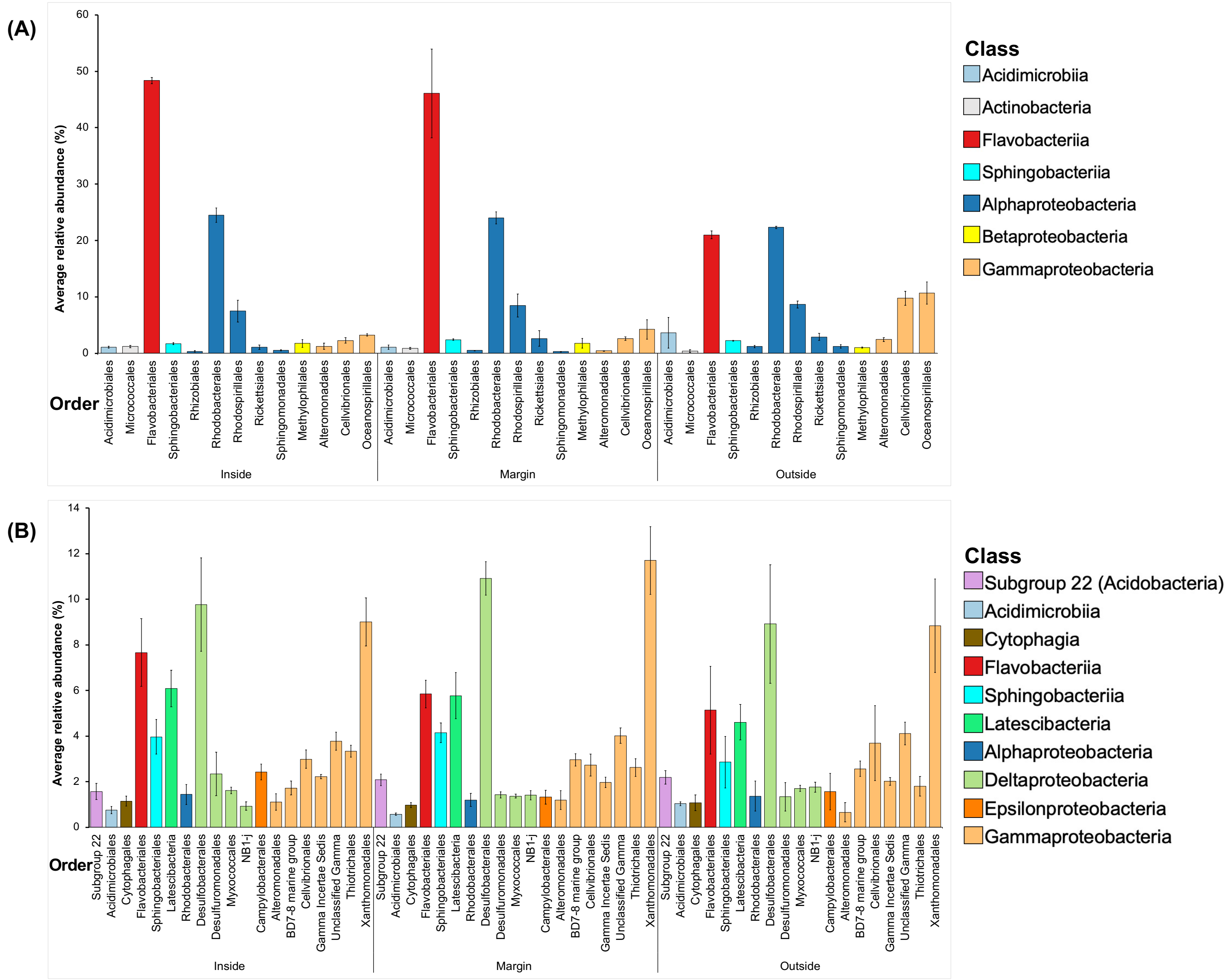

Fig. S2. Relative abundances of major microbial taxa, which were derived from two sample types: seawater **(A)**, sediment **(B)**. Sediment and sweater samples were obtained from three sampling points (inside, margin and outside). The OTUs were grouped into taxonomic categories (order), and each column was color-coded according to taxonomic class. The orders with a mean abundance of one percent (≥1%) are shown here. The bars represent the standard error of the mean.

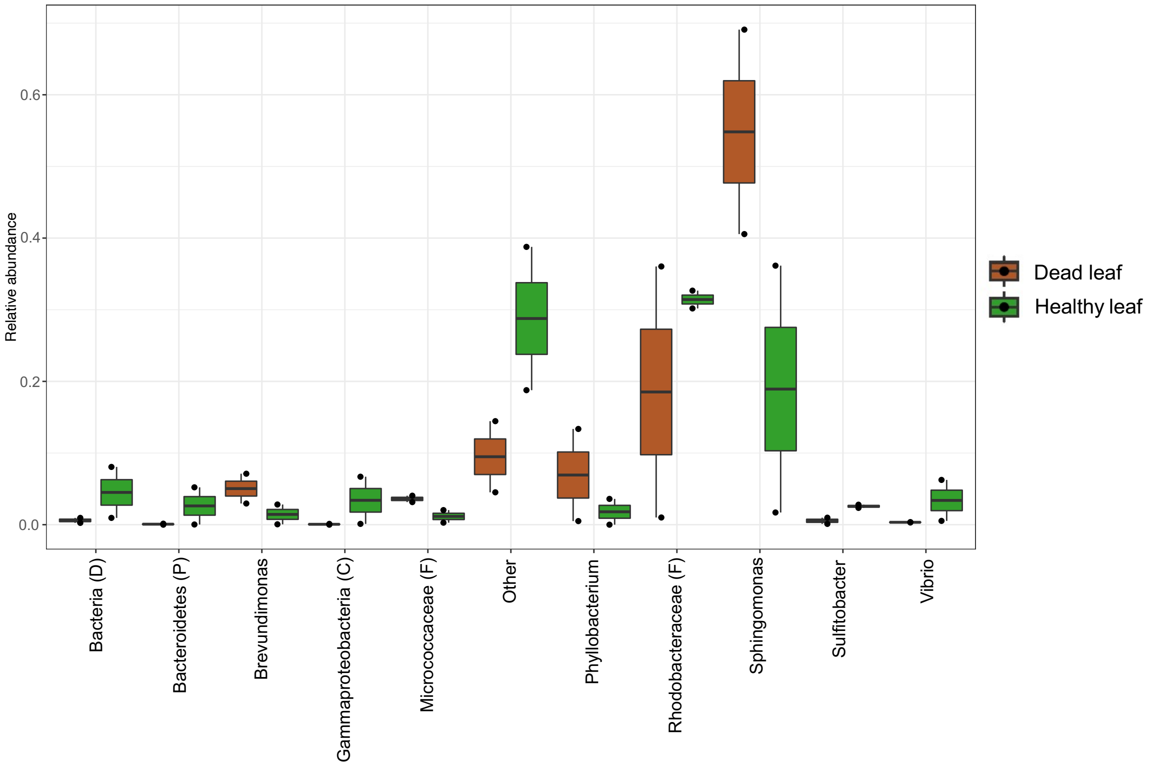

Fig. S3. Box plot illustrating differences in relative abundances of top 10 bacterial genera between dead leaves and healthy leaves. The OTUs not defined at the genus level were shown using the most specific taxonomic level available followed by a letter in brackets. Each letter represents a respective taxonomic level: D, Domain; P, Phylum, C, class; F, Family.

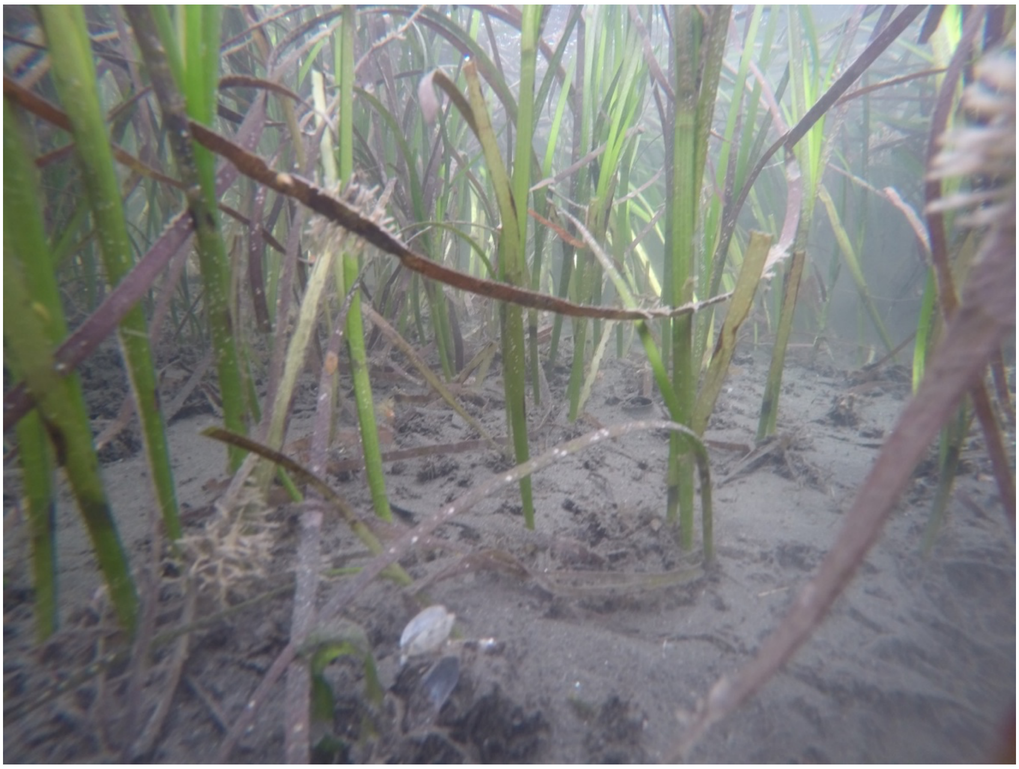

Fig. S4. A marine camera captured an under-water image of Futtsu eelgrass. The bottom surface looks more like sandy ground than sedimentary deposit, and there is little leaf litter lying on the eelgrass bottom.
